# Food insecurity in households with children and adolescents under the intersectionality between gender and race/skin color

**DOI:** 10.1590/0102-311XEN076325

**Published:** 2025-12-01

**Authors:** Isadora Dalla Valle Constantino Miguel, Alcenir Tavares Valente, Beatriz Picanço Bezerra de Menezes Costa, Isadora Rodrigues Gongô, Karina Andrade dos Reis Ferreira, Rafaella Peres da Costa, Rhuanna Laurent Silva Ribeiro, Sophia Santos de Castro Loureiro, Dirce Maria Lobo Marchioni, Jackeline Christiane Pinto Lobato, Valéria Troncoso Baltar

**Affiliations:** 1 Faculdade de Medicina, Universidade Federal Fluminense, Niterói, Brasil.; 2 Faculdade de Saúde Pública, Universidade de São Paulo, São Paulo, Brasil.; 3 Instituto de Saúde Coletiva, Universidade Federal Fluminense, Niterói, Brasil.

**Keywords:** Food Insecurity, Hunger, Race Factors, Family Structure, Insegurança Alimentar, Fome, Fatores Raciais, Estrutura Familiar, Inseguridad Alimentaria, Hambre, Factores Raciales, Estructura Familiar

## Abstract

Food insecurity refers to the lack of reliable access to quality food necessary for survival and well-being. It affects one’s physical, emotional, and cognitive health across different life stages. This study aims to estimate the prevalence of food insecurity in Brazilian households with no children/adolescents, one child/adolescent, or two or more children/adolescents, considering intersections of race/skin color, gender, and socioeconomic and demographic characteristics. We used data from 57,920 households from the 2017-2018 *Brazilian Household Budget Survey*. Perceived food insecurity at the household level was assessed using the *Brazilian Food Insecurity Scale* (EBIA, acronym in Portuguese), classifying households as food secure or experiencing any level of insecurity. A generalized linear regression model for complex sampling data, with binomial distribution and logarithmic link function, was adopted to verify the association between multiple variables and food insecurity prevalence. Overall, 36.7% (95%CI: 35.9; 37.5) of households experienced food insecurity with prevalence increasing as the number of children or adolescents rose (39%, 95%CI: 37.7; 40.2 in households with one child/adolescent and 51.3%, 95%CI: 50.0; 52.6 in those with two or more). Households earning less than 0.5 minimum wage had a food insecurity prevalence 3.4 times higher (95%CI: 3.2; 3.7) than those earning over two minimum wage. Food insecurity was also more prevalent in households headed by black/mixed-race individuals and when the number of children/adolescents was higher - except among black/mixed-race women, who experienced high food insecurity levels regardless of household composition. This study reinforces the deeply social nature of this public health issue and the urgent need for public policies to reduce social inequalities and structural racism in Brazil.

## Abstract (pt)

A insegurança alimentar refere-se à falta de acesso adequado a alimentos de qualidade necessários para a sobrevivência e o bem-estar. Insegurança alimentar afeta a saúde física, emocional e cognitiva em diferentes fases da vida. Este estudo tem como objetivo estimar a prevalência de insegurança alimentar em domicílios brasileiros sem crianças/adolescentes e com uma criança/adolescente ou duas ou mais crianças/adolescentes, considerando as interseções de raça/cor da pele, gênero e características socioeconômicas e demográficas. Utilizou-se dados de 57.920 domicílios da *Pesquisa de Orçamentos Familiares* de 2017-2018. A insegurança alimentar percebida em nível domiciliar foi avaliada usando a *Escala Brasileira de Insegurança Alimentar* (EBIA), classificando os domicílios como em segurança ou com algum nível de insegurança. Para verificar associações entre múltiplas variáveis e a prevalência de insegurança alimentar, adotou-se modelo de regressão linear generalizada para dados de amostragem complexa, com distribuição binomial e função de ligação logarítmica. De modo geral, 36,7% (IC95%: 35,9; 37,5) dos domicílios apresentaram insegurança alimentar, com aumento da prevalência à medida que o número de crianças ou adolescentes aumentava (39%, IC95%: 37,7; 40,2 nos domicílios com uma criança/adolescente e 51,3%, IC95%: 50,0; 52,6 naqueles com dois ou mais crianças/adolescentes). Os domicílios com renda inferior a 0,5 salário mínimo apresentaram prevalência de insegurança alimentar 3,4 vezes maior (IC95%: 3,2; 3,7) do que aqueles com renda superior a dois salários mínimos. A insegurança alimentar também foi mais prevalente em domicílios chefiados por indivíduos pretos/pardos e quando o número de crianças/adolescentes era maior - exceto entre mulheres pretas/pardas, que apresentaram altas prevalências de insegurança alimentar, independentemente da composição do domicílio. Este estudo reforça a natureza profundamente social desse problema de saúde pública e a necessidade urgente de políticas públicas para reduzir as desigualdades sociais e o racismo estrutural no Brasil.

## Abstract (es)

La inseguridad alimentaria consiste en la falta de acceso fiable a alimentos de calidad necesarios para la supervivencia y el bienestar. Afecta a la salud física, emocional y cognitiva de las personas en diferentes etapas de la vida. El objetivo de este estudio es estimar la prevalencia de la inseguridad alimentaria en hogares brasileños sin niños/adolescentes, con un niño/adolescente o con dos o más niños/adolescentes, teniendo en cuenta las intersecciones de raza/color de piel, género y características socioeconómicas y demográficas. Se utilizaron datos de 57.920 hogares de la *Encuesta de Presupuestos Familiares* de Brasil 2017-2018. La inseguridad alimentaria percibida a nivel de los hogares se evaluó utilizando la *Escala Brasileña de Inseguridad Alimentaria* (EBIA), que clasifica a los hogares como en seguridad desde el punto de vista alimentario o que experimentan algún nivel de inseguridad. Se adoptó un modelo de regresión lineal generalizado para datos de muestreo complejos, con distribución binomial y función de enlace logarítmica, con el fin de verificar la asociación entre múltiples variables y la prevalencia de la inseguridad alimentaria. En general, el 36,7% (IC95%: 35,9; 37,5) de los hogares experimentaron inseguridad alimentaria, con una prevalencia que aumentaba a medida que aumentaba el número de niños o adolescentes (39%, IC95%: 37,7; 40,2 en los hogares con un niño/adolescente y 51,3%, IC95%: 50,0; 52,6 en los que tenían dos o más). Los hogares con ingresos inferiores a 0,5 del salario mínimo tenían una prevalencia de inseguridad alimentaria 3,4 veces mayor (IC95%: 3,2; 3,7) que los que ganaban más que dos salarios mínimos. La inseguridad alimentaria también era más frecuente en los hogares encabezados por personas negras o mestizas y cuando el número de niños o adolescentes era mayor, excepto entre las mujeres negras o mestizas, que experimentaban altas prevalencias de inseguridad alimentaria independientemente de la composición del hogar. Este estudio refuerza la naturaleza profundamente social de este problema de salud pública y la urgente necesidad de políticas públicas para reducir las desigualdades sociales y el racismo estructural en Brasil.

## Introduction

Food security is characterized as the consolidation of the right of all people to regular, permanent, and unrestricted access to sufficient quality food for a healthy life, without compromising access to other basic needs, and respecting cultural traditions ^1^. Food insecurity, in turn, occurs when food security is not fully ensured; that is, when individuals lack reliable access to sufficient quality food necessary for survival ^1^. This condition profoundly affects relationships, work, education, and health ^2^.

A loss of food security, whether due to reduced meal frequency or poor meal quality, can result in inadequate nutrient consumption, which may compromise children’s cognitive functioning and overall development. Furthermore, this decline can cause stress and anxiety within families, affecting parents’ and children’s health ^3^ ^,^ ^4^. Adolescence is also a crucial stage of growth and development, marked by numerous physiological, hormonal, cognitive, and emotional changes ^5^.

Therefore, a balanced, nutrient-rich diet is crucial for preserving one’s physical, emotional, and cognitive health. Inadequate dietary patterns are associated with growth delays, severe nutritional, intellectual, and emotional impairments, as well as overweight, obesity, and several chronic diseases that extend into adulthood ^6^.

In Brazil, households with children and adolescents are more vulnerable to food insecurity ^6^ ^,^ ^7^ ^,^ ^8^. The burden of childcare and domestic responsibilities, historically assigned to women, significantly contributes to food insecurity, as the gendered division of labor restricts women’s participation in paid work ^8^ ^,^ ^9^ ^,^ ^10^ ^,^ ^11^. 

Crenshaw ^12^ first introduced the concept of intersectionality within the framework of black feminism, emphasizing how gender and race interact to maintain systemic oppression. Moderate and severe food insecurity are more prevalent in households headed by self-identified black women, underscoring the role of racial and gender inequality as structural determinants of food security in Brazil, beyond the influence of income ^13^.

This study aims to estimate the prevalence of food insecurity in Brazilian households without children/adolescents, with one child/adolescent, or two or more children/adolescents, considering the intersections of race/skin color, gender, and socioeconomic and demographic characteristics.

## Methods

This cross-sectional study uses a complex and representative sample of the Brazilian population: 57,920 households from the 2017-2018 *Brazilian Household Budget Survey* (POF, acronym in Portuguese), conducted by the Brazilian Institute of Geography and Statistics (IBGE, acronym in Portuguese). POF collects and disseminates data on household budget composition, living conditions, and quality of life ^14^.

The 2017-2018 POF edition was the first to use the *Brazilian Food Insecurity Scale* (EBIA, acronym in Portuguese) in its “Quality of Life” module. EBIA is a validated Brazilian scale adaptation of the Cornell indicator, developed in the United States, which measures perceived household food insecurity and hunger ^15^. To qualitatively describe the Brazilian food security profile, EBIA consists of 14 questions answered by the head of the consumption unit, considering the previous three months, and addressing food access and related hardships. The questions cover all consumption unit residents, listing them as over or under 18 years of age. Based on a standardized scoring system, each consumption unit is classified per food security status, with different cutoff points depending on the presence/absence of individuals under 18 years of age. In IBGE’s available data, EBIA classifies households into four categories: food security, mild food insecurity, moderate food insecurity, and severe food insecurity. In this study, households were classified as experiencing food security or food insecurity (at any level) ^14^.

POF collects information from EBIA and other income-related variables over a 12-month period. This approach ensures balanced seasonal coverage, minimizing the influence of short-term economic fluctuations. Moreover, 2018 represents a pre-crisis period, prior to the economic impacts of the COVID-19 pandemic, when Brazil had not yet been strongly affected by inflation or by government assistance programs, which have become more prominent in recent years. Thus, the data reflect the structural nature of food insecurity in Brazilian households.

Households were characterized by urban/rural location and Brazilian macroregions (North, Northeast, Central-West, Southeast, and South). We estimated the total number of residents and those up to 19 years old, classifying households as having no children/adolescents, 1 child/adolescent, or 2+ children/adolescents. Households were also classified by number of residents (up to 5 people, 6+). Gender (male, female) and race/skin color of the household head (white, black/mixed-race, Indigenous, or Asian) were used to compose the intersectionality variable: white man, black/mixed-race man, white woman, black/mixed-race woman, other). Due to limited representativeness in the POF, the “other” category was retained for sample expansion but was not used to interpret the results. Additional variables included participation of the head of household in paid or unpaid work during the reference 12 months (yes, no), years of schooling (< 1, 1-7, 8+), literacy (yes, no), household per capita income (< 0.5, 0.5-1, 1-2, > 2 minimum wages), and household composition (1 adult, 2+ adults).

### Statistical data analysis

The prevalence of food insecurity was calculated for each category of the study variables, with prevalence estimates and 95% confidence intervals (95%CI) used to describe data. A generalized linear regression model for complex sampling data, with binomial distribution and logarithmic link function, was adopted to verify the association between multiple variables and food insecurity prevalence ^16^. Variables were sequentially introduced according to those listed in Table 1, and all interactions involving intersectionality and the number of children/adolescents in the household were tested. A 5% significance level was adopted. The final model includes years of schooling, household per capita income, macroregion, and the interaction between intersectional categories of gender and race/skin color and the number of children/adolescents. All statistical analyses were performed in the R statistical package (http://www.r-project.org), using the *survey* library to consider the complex design of the POF sample ^17^.

**Table 1:** Prevalence of food insecurity by number of children and/or adolescents in households and intersectionality between gender and race/skin color, socioeconomic and demographic characteristics. 2017-2018 *Brazilian Household Budget Survey*.

	Number of children/adolescents in the household		
	None (n = 9,885,404)	1 (n = 6,818,088)	2+ (n = 8,571,953)
	% (95%CI)	% (95%CI)	% (95%CI)
Gender of household head			
Man	26.1 (25.0; 27.2)	34.4 (32.9; 36.0)	46.5 (44.9; 48.0)
Woman	31.8 (30.5; 33.1)	45.4 (43.6; 47.1)	58.3 (56.4; 60.2)
Race/Skin color of household head			
White	20.3 (19.3; 21.4)	29.8 (28.0; 31.6)	38.2 (36.1; 40.4)
Black/Mixed-race	37.0 (35.8; 38.2)	46.2 (44.7; 47.8)	58.5 (57.0; 59.9)
Asian/Indigenous/Undeclared	19.2 (14.6; 24.9)	29.2 (20.8; 39.1)	49.1 (37.8; 60.6)
Intersectionality			
White man	18.0 (16.8; 19.3)	25.8 (23.6; 28.2)	33.8 (31.3; 36.4)
Black/Mixed-race man	34.4 (32.8; 36.0)	41.5 (39.5; 43.5)	54.3 (52.3; 56.2)
White woman	23.4 (21.8; 25.2)	35.8 (32.9; 38.9)	46.0 (42.6; 49.5)
Black/Mixed-race woman	40.6 (38.9; 42.4)	52.4 (50.3; 54.5)	63.9 (61.8; 65.9)
Other	19.2 (14.6; 24.9)	29.2 (20.8; 39.1)	49.1 (37.8; 60.6)
Macroregion			
Southeast	25.1 (23.6; 26.6)	33.3 (31.0; 35.8)	44.2 (41.6; 46.9)
South	15.2 (13.7; 16.8)	22.4 (20.1; 24.8)	33.6 (30.8; 36.5)
North	44.8 (42.0; 47.6)	57.3 (53.9; 60.6)	68.4 (65.8; 71.0)
Northeast	41.1 (39.6; 42.5)	52.2 (50.4; 54.0)	63.4 (61.6; 65.2)
Central-West	29.7 (27.0; 32.6)	37.1 (34.1; 40.3)	44.4 (40.7; 48.1)
Area			
Urban	27.7 (26.7; 28.6)	37.4 (36.0; 38.7)	49.1 (47.6; 50.6)
Rural	34.3 (32.4; 36.2)	49.8 (47.1; 52.5)	62.2 (59.8; 64.5)
Head of household had paid/unpaid work in the past 12 months			
Yes	28.3 (27.4; 29.2)	38.6 (37.3; 39.9)	51.2 (49.8; 52.5)
No	38.8 (34.8; 43.0)	49.5 (44.4; 54.7)	55.0 (49.7; 60.3)
No information	74,488	100,688	171,825
Household head can read and write			
Yes	26.4 (25.5; 27.4)	37.4 (36.1; 38.7)	49.6 (48.2; 50.9)
No	45.2 (42.9; 47.4)	61.4 (57.9; 64.8)	71.4 (68.0; 74.6)
Years of schooling of the household head			
< 1	42.0 (39.5; 44.4)	57.8 (53.7; 61.8)	71.5 (67.3; 75.4)
1-7	33.8 (32.5; 35.2)	48.6 (46.5; 50.7)	60.0 (58.0; 62.0)
8+	22.8 (21.7; 23.9)	33.7 (32.2; 35.2)	45.0 (43.3; 46.6)
Household composition (adults)			
1	32.3 (30.4; 34.3)	41.0 (26.1; 57.8)	14.6 (1.06; 73.3)
2+	27.7 (26.8; 28.6)	39.0 (37.7; 40.2)	51.3 (50.0; 52.6)
Number of people in the household			
Up to 5	28.4 (27.6; 29.3)	38.8 (37.6; 40.1)	48.0 (46.6; 49.4)
6+	48.9 (36.4; 61.5)	45.9 (38.7; 53.3)	64.6 (61.9; 67.2)
Per capita household income (minimum wages)			
< 0.5	61.0 (57.0; 64.8)	64.7 (61.9; 67.3)	73.5 (71.9; 75.0)
0.5-1	50.8 (48.5; 53.1)	50.9 (48.8; 52.9)	51.9 (49.7; 54.0)
1-2	34.0 (32.7; 35.4)	35.2 (33.2; 37.3)	34.7 (32.1; 37.3)
> 2	14.8 (13.8; 15.9)	15.7 (13.8; 17.8)	14.7 (12.3; 17.4)

95%CI: 95% confidence interval.

### Ethical consideration

This study was conducted according to the guidelines laid down in the *Declaration of Helsinki*. This study used secondary data from the 2017-2018 POF, conducted by the IBGE. This study used publicly available data from IBGE’s POF databases, in which it is impossible to identify individual respondents. As the data are anonymized and released for public use by IBGE, no ethical approval was required for this study, in accordance with *Resolution n. 510/2016* of the Brazilian National Research Ethics Commission (CONEP, acronym in Portuguses), which exempts research using publicly available data from review.

## Results

In Brazil, during 2017-2018, the prevalence of food insecurity was 36.7% (95%CI: 35.9; 37.5), with a significant difference between households with no children/adolescents (28.5%, 95%CI: 27.6; 29.4), with 1 child/adolescent (39%, 95%CI: 37.7; 40.2), and with 2+ children/adolescents (51.3%, 95%CI: 50.0; 52.6).

Table 1 shows that the highest food insecurity prevalence was recorded in the North and Northeast, rural areas, households headed by individuals without paid work in the previous 12 months, households whose heads are illiterate or less educated, and households with 6+ residents. In all these conditions, food insecurity prevalence is even higher if the household has children/adolescents. Regarding family composition, food insecurity prevalence was higher in households with only 1 adult and up to 1 child/adolescent, as well as in larger households with multiple adults and 2+ children/adolescents (51.3%, 95%CI: 50.0; 52.6).

**Figure 1:**
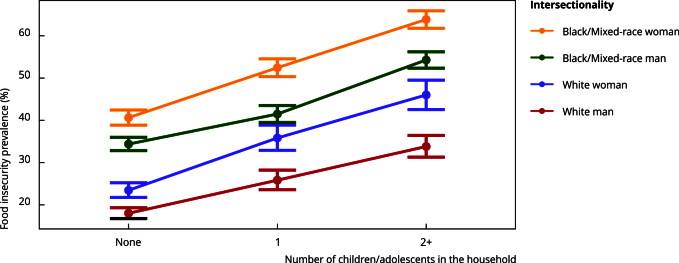
Prevalence of food insecurity in households by number of children/adolescents, race/skin color and household head’s gender intersectionality.

Figure 1 highlights the results (Table 1) for the variable representing the intersection of the household head’s race/skin color and gender. Households headed by self-declared black/mixed-race individuals have a higher food insecurity prevalence than those headed by self-declared white individuals, and the same occurs in households with female heads compared to men. This pattern reveals the combined effect of gender and race/skin color, with food insecurity prevalence increasing as the number of children/adolescents rises. The highest food insecurity prevalence was recorded in households headed by self-declared black/mixed-race women with 2+ children/adolescents (63.9%, 95%CI: 61.8; 65.9), while the lowest prevalence occurs in households headed by self-declared white men without children/adolescents (18%, 95%CI: 16.8; 19.3). In terms of per capita income, food insecurity prevalence was lowest in households with no children/adolescents and earning > 2 minimum wages (14.8%, 95%CI: 13.8; 15.9), and highest in households with 2+ children/adolescents and < 0.5 minimum wage per capita (73.5%, 95%CI: 71.9; 75.0).

Table 2 shows the results for the log-binomial linear regression model, which associates food insecurity prevalence with the household head’s years of schooling, household per capita income, macroregion of residence, and the interaction between gender, race/skin color of the head, and number of children/adolescents. The lower the educational level of the head of the household head, the higher the food insecurity prevalence. Keeping other variables of the model fixed, households headed by individuals with < 1 year of schooling had a 12% higher prevalence compared to those with 8+ years (prevalence ratio - PR = 1.12, 95%CI: 1.08; 1.16). Households with per capita < 0.5 minimum wage showed food insecurity prevalence of 3.4 times higher than those with > 2 minimum wages (95%CI: 3.2; 3.7). The North and Northeast recorded the highest prevalence, with the North showing a prevalence 14% higher than the Southeast (PR = 1.14, 95%CI: 1.1; 1.2).

**Table 2:** Results of the log-binomial regression model for the prevalence of food insecurity per sociodemographic characteristics and the intersectionality between gender, race/skin color, and number of children or adolescents in the households. 2017-2018 *Brazilian Household Budget Survey*.

			PR	95%CI	p-value
Years of schooling of the household head					
< 1/8+			1.12	1.08; 1.16	< 0.01
1-7/8+			1.09	1.06; 1.12	< 0.01
Per capita household income (minimum wages)					
< 0.5/> 2			3.41	3.18; 3.65	< 0.01
< 0.5-1/> 2			2.77	2.59; 2.96	< 0.01
1-2/> 2			2.05	1.92; 2.18	< 0.01
Macroregions					
Southeast/South			1.36	1.27; 1.47	< 0.01
Central-West/South			1.45	1.37; 1.57	< 0.01
Northeast/South			1.55	1.45; 1.66	< 0.01
North/South			1.67	1.55; 1.79	< 0.01
Interaction gender/race/skin color and number of children/adolescents					< 0.01
Race/Skin color contrast					
Black/Mixed race vs. white	Man	None	1.37	1.19; 1.56	< 0.01
Black/Mixed race vs. white	Man	1	1.17	1.01; 1.36	0.02
Black/Mixed race vs. white	Man	2+	1.18	1.05; 1.33	< 0.01
Black/Mixed race vs. white	Woman	None	1.19	1.04; 1.37	< 0.01
Black/Mixed race vs. white	Woman	1	1.08	0.94; 1.24	0.99
Black/Mixed race vs. white	Woman	2+	1.04	0.93; 1.17	0.99
Gender contrast					
Woman vs. man	White	None	1.30	1.11; 1.54	< 0.01
Woman vs. man	Black/Mixed-race	None	1.14	1.04;1.25	< 0.01
Woman vs. man	White	1	1.28	1.06; 1.54	< 0.01
Woman vs. man	Black/Mixed-race	1	1.17	1.07;1.28	< 0.01
Woman vs. man	White	2+	1.24	1.07; 1.43	< 0.01
Woman vs. man	Black/Mixed-race	2+	1.09	1.02; 1.17	< 0.01
Number of children/adolescents contrast					
1 vs. none	Man	White	1.16	0.97; 1.39	0.45
2+ vs. 1	Man	White	1.10	0.93; 1.29	< 0.01
2+ vs. none	Man	White	1.27	1.09; 1.48	< 0.01
1 vs. none	Woman	White	1.14	0.96; 1.35	0.99
2+ vs. 1	Woman	White	1.06	0.90; 1.25	0.99
2+ vs. none	Woman	White	1.21	1.03; 1.41	< 0.01
1 vs. none	Man	Black/Mixed-race	1.00	0.91; 1.10	0.99
2+ vs. 1	Man	Black/Mixed-race	1.10	1.02; 1.20	< 0.01
2+ vs. none	Man	Black/Mixed-race	1.10	1.00; 1.20	0.02
1 vs. none	Woman	Black/Mixed-race	1.02	0.93; 1.12	0.99
2+ vs. 1	Woman	Black/Mixed-race	1.03	0.95; 1.11	0.99
2+ vs. none	Woman	Black/Mixed-race	1.05	0.97; 1.15	0.99

95%CI: 95% confidence interval; PR: prevalence ratio.

The interaction between race/skin color, gender, and the number of children/adolescents in the household suggests that the pattern of higher food insecurity prevalence with more children/adolescents is not consistent across the demographic groups. When comparing households with 2+ child/adolescents to those without, the increase in food insecurity prevalence is around 20% among white heads of households (1.27, 95%CI: 1.09; 1.48 for men; 1.21, 95%CI: 1.03; 1.41 for women). Among black/mixed-race heads, the increase is around 10% for men (1.1; 95%CI: 1.0; 1.2) and not statistically significant for women (1.05, 95%CI: 0.97; 1.15).

## Discussion

Food insecurity prevalence was high throughout Brazil in 2017-2018 and was associated with family composition, as well as socioeconomic and demographic characteristics. Our result suggests that food insecurity prevalence increases with the number of children/adolescents in the household. However, this relationship varies by the gender and race/skin color of the heads of households, with notable differences between men and women and between white or black/mixed-race heads. As expected, household per capita income is strongly associated with food insecurity prevalence. These findings highlight the strong social determinant underlying this public health issue.

To our knowledge, this is the first study to suggest this intersectional relationship between the household head’s race/skin color, gender, and the number of children/adolescents in relation to food insecurity prevalence. Hirata ^18^ analyzes the social, racial, and gendered division of care labor, highlighting how domestic and caregiving work remains predominantly unpaid and performed by women. Consequently, households headed by women with children/adolescents - as shown in this study - exhibit a higher food insecurity prevalence. Rodrigues et al. ^11^ observed a high prevalence of severe food insecurity in female-headed, low-income urban households participants of cash transfer welfare programs. Women’s disproportionate share of care labor limits access to paid employment and may explain these families’ vulnerability to food insecurity. 

Santos et al. ^13^ suggested an intersectional relationship between gender and race/skin color and the prevalence of moderate and severe food insecurity across all macroregions of Brazil. However, the presence of children in the household was treated only as a control variable. The authors classified household heads into combinations of gender and race/skin color: white man/woman, mixed-race man/woman, and black man/woman. As in this study, Indigenous and Asian categories were excluded due to their small representation within POF ^13^. Santos et al. ^8^ observed that households with children had higher rates of moderate/severe food insecuruty, particularly when headed by black/mixed-race individuals.

Thus, this study expands on the intersectionality of gender and race/skin color described by Santos et al. ^13^ by incorporating an additional dimension of family composition. The findings suggest that food insecurity prevalence is lower in households without children/adolescents and increases with the number of children/adolescents. Moreover, this higher prevalence is not uniform across different categories of the intersectionality of race/skin color and gender.

The lack of association between the number of children/adolescents and food insecurity prevalence in households headed by black/mixed-race women may reflect the already elevated levels of food insecurity in this group, regardless of household composition. This reflects the historical racism, sexism, and oppression experienced by black/mixed-race women. Other groups, to varying degrees, have lower food insecurity levels. Thus, a greater number of children/adolescents in the household is strongly associated with higher food insecurity prevalence. In absolute terms, it is concerning that households headed by women consistently exhibit higher food insecurity prevalence than those headed by men across all categories of children/adolescents.

In 2021, IBGE released the results of the Gender Statistics study, showing that, in Brazil, women spend on average almost twice as many hours per week on household chores as men, with black/mixed-race women spending on average 1.6 hours more than white women. Furthermore, the study found that men’s participation in the labor market is 19.9% higher than women’s. Informality is higher among women and even higher among black/mixed-race women. Furthermore, women’s income was equivalent to only 63.3% of men’s income, and black/mixed-race women represent the most vulnerable group among women ^19^. The same study revealed that the proportion of women employed in part-time work in the North and Northeast exceeds that of other regions, which is higher for women than men in all macroregions ^19^. These findings align with the analyses of this study, which showed a higher food insecurity prevalence in households headed by women who self-declare as black/mixed-race, reflecting their greater vulnerability.

Based on Nancy Fraser’s concept of gender justice, food insecurity results from economic, social, and political injustices. Capitalism, structural inequalities, and the lack of social representation for the most vulnerable populations in political spaces reinforce vulnerability to food insecurity ^10^. A systematic review ^20^ identified income as the central determinant of severe food insecurity. The authors proposed a conceptual model describing the relationship between social indicators (monthly income, educational level, and household characteristics) and food insecurity; a relationship mediated by income; and a relationship mediated by others social indicators, such as the employment status and educational level of the head of household, along with income ^20^. Households headed by women or black/mixed-race individuals tend to have lower educational levels, resulting in lower family income ^20^.

This study has some limitations. The sex variable was used as a proxy for gender, without considering the diversity of gender identities. Moreover, due to low representation in the POF sample, other minorities - such as Indigenous, Asian, and undeclared individuals - were grouped into a single category, regardless of gender. However, the POF’s national scope enables the analysis of second-degree interactions between variables of interest: sex/gender, race/skin color, and number of children/adolescents in the household. Although it was impossible to consider Indigenous and Asian groups, the results for white and black/mixed-race individuals are relevant for a reliable portrait of food insecurity severity, especially in the most vulnerable households headed by black/mixed-race women. A key strength of this study is that the observed associations persisted independently of household per capita income, which was accounted for in the model.

Although the association between social factors and food insecurity prevalence is well documented in the scientific literature and reaffirmed by this study, there remains a lack of research specifically addressing the effects of the intersectionality of gender and race/skin color on food insecurity. Few studies address both race/skin color and gender simultaneously in relation to food insecurity. This is the first study to report second-degree intersectionality, including household composition regarding children/adolescents. Future research should further investigate this synergistic effect among black/mixed-race women and households with dependents under 19 years of age in relation to higher food insecurity prevalence. Together, these three characteristics contribute to the magnitude of food insecurity, reaching alarming and unacceptable levels among affected households.

This study analyzed geographic and socioeconomic factors, highlighting the presence of children/adolescents in households as a crucial factor in worsening food insecurity conditions. In 2010, the Municipal Human Development Index (M-HDI) values of Brazilian macroregions were 0.667 for the North, 0.663 for the Northeast, 0.757 for the Central-West, 0.766 for the Southeast, and 0.754 for the South; with the North and Northeast remaining in the medium range of the “M-HDI income” category, while other macroregions fell within the high range ^21^. Therefore, given the historical social inequalities across geographic macroregions, this study highlights discrepancies regarding access to quality food, with food insecurity prevalence being more severe in the North, followed by the Northeast, the Central-West, the Southeast, and the South. This disparity is further aggravated by the presence of children and adolescents in the household.

The fact that the North-Northeast axis has the lowest M-HDI in the education and income categories ^21^ and the highest food insecurity prevalence reveals that the household head’s level of education and household per capita income directly reflects food security status. Income is also associated with the macroregion, since the most vulnerable and poorest macroregions coincide with those with the highest food insecurity prevalence. Furthermore, higher educational levels of the household head were associated with lower food insecurity prevalence. Consistent with the results of this study, other studies have shown the association between food security and educational attainment. For instance, in Togo (Africa), a severe food insecurity rate of 46.3% was observed among individuals over 15 years old who attended only primary school or did not complete it ^22^. Additionally, 5% of the total effect of gender disparity in food insecurity is mediated by the household head’s educational level ^23^. These findings clearly indicate that access to quality education, promoting basic literacy skills, and securing a position in the job market - a condition strongly related to educational level - are essential to overcome food insecuruty at a national level.

## Conclusion

The high prevalence of food insecurity in Brazil is closely associated with household income and the number of children/adolescents residing in the household. Moreover, this relationship varies according to the gender and self-declared race/skin color of the household head, with notable differences between male- and female-headed households, as well as between those identifying as white or black/mixed-race. These findings highlight the deeply social nature of this public health issue and underscore the urgent need for public policies aimed at reducing social inequalities and addressing structural racism in Brazil.

## Data Availability

The sources of information used in the study are indicated in the body of the article.
